# Semantic-based crossmodal processing during visual suppression

**DOI:** 10.3389/fpsyg.2015.00722

**Published:** 2015-06-02

**Authors:** Dustin Cox, Sang Wook Hong

**Affiliations:** Department of Psychology, College of Science, Florida Atlantic University, Boca Raton, FL, USA

**Keywords:** multisensory integration, semantic processing, continuous flash suppression, visual awareness, semantic priming

## Abstract

To reveal the mechanisms underpinning the influence of auditory input on visual awareness, we examine, (1) whether purely semantic-based multisensory integration facilitates the access to visual awareness for familiar visual events, and (2) whether crossmodal semantic priming is the mechanism responsible for the semantic auditory influence on visual awareness. Using continuous flash suppression, we rendered dynamic and familiar visual events (e.g., a video clip of an approaching train) inaccessible to visual awareness. We manipulated the semantic auditory context of the videos by concurrently pairing them with a semantically matching soundtrack (congruent audiovisual condition), a semantically non-matching soundtrack (incongruent audiovisual condition), or with no soundtrack (neutral video-only condition). We found that participants identified the suppressed visual events significantly faster (an earlier breakup of suppression) in the congruent audiovisual condition compared to the incongruent audiovisual condition and video-only condition. However, this facilitatory influence of semantic auditory input was only observed when audiovisual stimulation co-occurred. Our results suggest that the enhanced visual processing with a semantically congruent auditory input occurs due to audiovisual crossmodal processing rather than semantic priming, which may occur even when visual information is not available to visual awareness.

## Introduction

The objects and events we encounter in everyday life are often experienced in multiple sensory modalities. Multisensory integration can enrich perceptual experience of objects and events by enhancing the saliency of stimuli (Stein and Stanford, 2008; Evans and Treisman, 2010). The advantages of multisensory integration have been evidenced by faster response times (RTs) in speeded classification tasks when auditory pitch and visual elevation are congruent with each other (Bernstein and Edelstein, 1971; Ben-Artzi and Marks, 1995), improved visual motion perception with congruent auditory information (Cappe et al., 2009; Lewis and Noppeney, 2010), and enhanced speech perception with synchronous audiovisual inputs (Pandey et al., 1986; Plass et al., 2014).

Multisensory congruency generally indicates that multiple unimodal stimuli are present closely in space or time. Multisensory spatiotemporal congruency often results in enhancement of behavioral and perceptual performances (Stein et al., 1988). Auditory and visual stimuli that have spatial alignment can generate more efficient saccadic eye movements to the target in non-human primates (Bell et al., 2005). Human saccadic eye movements are also faster toward visual targets when auditory and visual stimuli have closer spatiotemporal proximity (Frens et al., 1995). The detection of unimodal objects and events can be enhanced by a spatially and/or temporally co-occurring stimulus in another modality (Vroomen and de Gelder, 2000; Lovelace et al., 2003; Bolognini et al., 2005; Noesselt et al., 2008).

Semantic congruency is also considered to be an important factor that determines multisensory integration (Doehrmann and Naumer, 2008; Spence, 2011). Audiovisual crossmodal semantic congruency effects have been examined by testing whether behavioral performance is enhanced by pairing an auditory stimulus and a visual stimulus that match or mismatch in meaning, such as pairing the sound of a dog barking with an image of a dog or cat (Laurienti et al., 2003; Hein et al., 2007). Participants tend to be faster and/or more accurate when identifying visual stimuli paired with auditory stimuli that have a semantically congruent than incongruent relationship (Laurienti et al., 2003, 2004; Iordanescu et al., 2008; Schneider et al., 2008; Chen and Spence, 2010).

The semantic content of auditory information can also affect visual awareness. The auditory semantic context of sounds heard during viewing of bistable figures can influence the predominance of a given percept (Hsiao et al., 2012). When viewing different dichoptic images during binocular rivalry, the dominance duration of a visual stimulus paired with a semantically congruent sound is significantly longer than when the same stimulus is paired with a semantically incongruent sound (Chen et al., 2011). Considering that perceptual dominance during binocular rivalry is dependent on the relative strength of dichoptically presented stimuli (Levelt, 1965), a longer period of dominance for an audiovisually congruent stimulus suggests that multisensory integration can strengthen a visual stimulus, resulting in the prolonged predominance of the stimulus. The longer predominance of a visual stimulus paired with a semantically congruent sound during binocular rivalry, however, cannot indicate whether the congruent sound influences the strength of the visual stimulus while it is suppressed from visual awareness. Multisensory integration may only occur when the congruent visual stimulus is dominantly perceived, and thus congruent auditory input might only exert an influence on dominance durations when visual stimuli are consciously perceived.

The possibility of multisensory integration based on semantic congruency when visual stimuli are suppressed from visual awareness has been supported by recent studies using continuous flash suppression (CFS; see Tsuchiya and Koch, 2005). CFS is a modification of binocular rivalry, in that, dynamically changing, highly salient “noise” patterns presented to one eye can suppress a stimulus presented to the other eye from visual awareness for extended periods of time. The measurement of the time of the breakup of CFS can indicate the relative strength of visual stimuli to gain access to the visual awareness of observers (Stein et al., 2011).

The results of two recent studies demonstrate that congruent semantic auditory information in addition to temporal congruency can enhance the processing of *dynamic* visual stimuli, which are suppressed from visual awareness (Alsius and Munhall, 2013; Plass et al., 2014). A dynamic talking face suppressed from visual awareness by CFS can break suppression and reach visual awareness quicker when the original (matched) soundtrack accompanies the lip movements of the face compared to a mismatched soundtrack pair (Alsius and Munhall, 2013). In another study, a dynamic talking face presented during CFS can speed up the identification of a spoken target word if the lip-movements of the face correspond synchronously (Plass et al., 2014). However, it is not clear whether this congruency effect on visual speech processing is mediated by purely semantic-based multisensory integration since the influence of audiovisual semantic congruency could not be separated from speech stimuli while fully controlling for audiovisual temporal synchrony during CFS.

In the current study, we examined whether *purely* semantic-based multisensory integration influenced access to visual awareness for familiar dynamic visual events while limiting spatiotemporal congruency. Using CFS, we measured participants’ RTs to identify suppressed visual events when participants simultaneously heard soundtracks that were either semantically congruent or incongruent with the visual events. The audiovisual events, such as a moving racecar and an approaching train, were chosen because there is a lesser amount of specific congruent timing between their constituent auditory and visual event components. We specifically hypothesize that audiovisual crossmodal integration occurs even when visual stimuli are suppressed from visual awareness, and thus, semantically congruent audiovisual events will break up suppression and will be perceived earlier than incongruent events. In a control experiment, we tested whether the semantic congruency effect occurs due to crossmodal semantic priming by presenting the soundtracks prior to the visual events. In an additional control experiment, we tested our hypothesis further using static images with which any residual spatiotemporal crossmodal correspondences were removed.

## Experiment 1

To determine whether auditory semantic information can influence visual awareness of events, we measured the latencies for participants to identify one of three (3AFC task) familiar visual events with concurrent soundtracks that were initially suppressed by CFS. The soundtracks varied in their semantic relationships to the videos so that they matched (congruent audiovisual soundtrack condition), mismatched (incongruent audiovisual soundtrack condition), or were silent (neutral video-only condition). If semantic auditory contexts affect visual processing of dynamic events, which are suppressed from visual awareness, there should be a difference in the RT for participants to become aware of event videos as they break CFS across the different soundtrack conditions. We expected that visual event videos that were semantically congruent with a concurrently heard soundtrack would break up suppression relatively sooner than when soundtracks were incongruent or neutral as indicated by faster RTs in the congruent audiovisual soundtrack condition.

### Method

#### Participants

Thirty-three (nine males) undergraduate students participated in Experiment 1 for course credit. The participants were naïve to the purpose of this study. All participants had normal or corrected-to-normal vision and normal hearing as indicated by self-report. All participants signed an informed consent form approved by the Florida Atlantic University Institutional Review Board before participating in this experiment.

#### Apparatus and Stimuli

The visual stimuli were presented on a Sony CPD-G520, 21′ CRT display (100 Hz frame rate). The presentation of stimuli and collection of response data was manipulated by the Psychophysics Toolbox (Brainard, 1997; Pelli, 1997) in Matlab (MathWorks). Visual stimuli were presented in a dark room to observers positioned 90 cm from the CRT monitor whose R, G, B guns were calibrated using a light meter (IL-1700) and a luminance meter (Minolta LS100), creating a linearized look-up-table (eight-bit for each R, G, and B guns). A four-mirror stereoscope was used to achieve dichoptic presentation of the visual stimuli characteristic of a binocular rivalry experiment. Auditory stimuli were presented using Acoustic Noise-Canceling headphones (Bose QuietComfort).

The visual stimuli used in Experiment 1 were three dynamic and familiar event video clips, one of which was presented to one eye of a participant in each trial. The three brief video clips (7 s in duration) were black and white and depicted an approaching train, a man playing guitar, or racecars circling a racetrack. The video clip stimuli were edited using iMovie. The video clips were presented within rectangular apertures (3.91° × 3.2°) created by black rectangular fusion contours (4.36° × 3.42°). Three audiovisual soundtrack conditions were tested. In the congruent audiovisual soundtrack condition, each video clip was presented with its original soundtrack (e.g., an approaching train with a train soundtrack). In the incongruent audiovisual soundtrack condition, each video clip was overdubbed with a soundtrack from one of the other two videos (e.g., an approaching train with a racecar soundtrack). In the neutral video-only condition, video clips were presented without any sound. The suppressors, dynamically changing Mondrian-like patterns, were presented to the other eye. Each suppressor was composed of 200 rectangular patches with random sizes. The luminance of each patch was randomly assigned, but within a predetermined range whose maximum and minimum values were used to compute the contrast of the suppressors. The mean luminance of the suppressors was fixed at 55 cd/m^2^, which was identical to the luminance of the background. Sixty Mondrian-like patterns were created and presented every 100 msec (10 Hz).

Calibration of the stereoscope was achieved by participants’ self-report of the vertical alignment of small nonius lines (0.04° × 0.22°) that extended from the center of the top and bottom of the inner edge of the rectangular image apertures presented to the left and right eye, respectively. The stereoscope was calibrated prior to the practice trial, and the calibration was checked again prior to the beginning of the experimental trial for each participant. Participants were also instructed to monitor the alignment of the nonius lines in between trials throughout the experiment and to inform the experimenter if they became misaligned.

### Procedure

Participants viewed a dichoptic presentation consisting of a dynamic Mondrian stimulus that was presented in one eye while the other eye was simultaneously presented with one of nine target event videos (three video conditions by three soundtrack conditions). The Mondrian stimuli served to initially suppress the target video that was concurrently presented to the opposite eye from visual awareness. The eye that viewed the target video in each condition was considered the target eye. Each target event video condition (train, guitar, racecar), soundtrack condition (congruent audiovisual, incongruent audiovisual, neutral video-only), and target eye condition (left, right) was counterbalanced and randomly presented eight times to each participant for a total of 144 trials.

**FIGURE 1:**
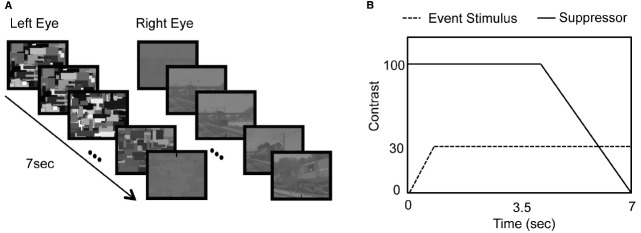
**(A)** Schematic diagram of stimulus presentation. **(B)** Changes in contrast of the suppressor (solid line) and the event stimulus (dashed line).

The relative luminance contrast in relation to the background for the Mondrian suppressors and target event videos was manipulated to ensure that the Mondrian stimuli achieved initial perceptual dominance followed by the breaking of suppression of the target video into perceptual dominance in each experimental trial (Yang et al., 2007). The target event videos were initially presented to one eye at 0% contrast before gradually increasing in contrast at equal increments over the first second of each experimental trial until reaching 30% contrast. In each trial, the Mondrian suppressor was initially presented at full (100%) contrast for the first 4 s before decreasing in contrast at equal decrements over the course of the remaining 3 s, so the Mondrian stimulus decreased in contrast to 0% by the end of the last 3 s of each 7-s trial duration (see Figure 1).

The participants’ task was to report which target event video was viewed in each trial. Three response keys located at the numeric keypad portion on the right side of a computer keyboard were designated (participants were instructed to press the “1” key if they saw the train video, the “2” key if they saw the guitar video, or the “3” key if they saw the racecar video) prior to beginning the practice trials that were completed before the actual experiment. Participants were reminded again of the response key assignments as needed throughout the practice trials and once more prior to beginning the experimental trials of Experiment 1. The elapsed time from the moment of pressing the spacebar on the computer keyboard, which initiated each trial, until the moment of pressing the “1,” “2,” or “3” key on the keyboard was recorded as a RT. Participants were instructed to respond only when confident about identification of the video. Participants were also encouraged to not guess or respond based on the soundtracks they heard since the soundtracks would not always be informative for determining the correct response in the trials.

Participants were familiarized with the task during a run of practice trials that were identical to the experimental trials of Experiment 1 but consisted of only 12 repetitions. Successful practice trial performance was indicated once each participant demonstrated correct memorization of the response keys and was based on consistently correct responding as determined by the experimenter.

### Results and Discussion

The data from 28 participants (seven males) were analyzed. We excluded five participants’ data that had overall average error rates greater than or equal to chance level responding (i.e., chance responding rate on a 3AFC = 0.33). A trial in which the response did not correspond with the actual video presented was considered to be an error. Error rates greater than chance are potentially indicative of a lack of motivation and/or understanding of the task, or a tendency to guess when responding. To ensure that the mean RT measurements were based only on correct responses, the RTs from incorrect response trials were excluded from the analysis. Trials where a video did not break up the suppression occurred when a key press response was not made during the 7-s duration of stimulus presentation. Since participants were encouraged to not guess the event video that was viewed, trials where no response was made were not considered to be incorrect, but they were also removed from the analysis.

A three by three (three event video conditions by three soundtrack conditions), two-way repeated measures ANOVA was conducted to examine the effect of the event viewed, the type of soundtrack heard, and the interaction between the event and soundtrack conditions on the mean RTs to discriminate the visual event videos. The analysis revealed a significant main effect of the event video viewed [*F*(2,26) = 10.058, *p* = 0.001, η^2^ = 0.271] and the type of soundtrack heard [*F*(2,26) = 10.263, *p* = 0.000, η^2^ = 0.275]. There was no significant interaction effect between the event and soundtrack conditions [*F*(4,24) = 0.808, *p* = 0.480, η^2^ = 0.029]. The significant main effect of the event video factor was not surprising since there were different amounts of luminance and motion information contained in the three videos. Differences in visual stimulus saliency may differentiate the time of the breakup of suppression. The lack of a significant interaction between sound and event conditions indicates a consistent effect of sound among the different events.

**FIGURE 2:**
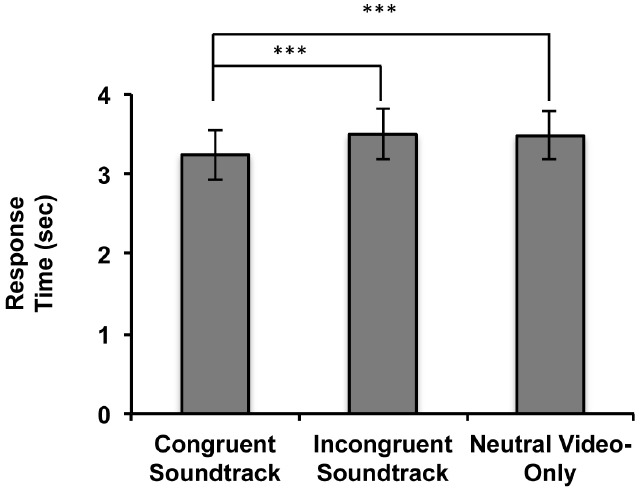
**Results of Experiment 1.** Response times (RTs: time of breakup of suppression) for the aggregated audiovisual soundtrack conditions in the 3AFC event video identification task when soundtracks were heard during event video viewing (***p ≤ 0.001). Error bars represent ± 1 standard error.

Since no significant interaction between sound and event conditions was found, we aggregated data based on the sound conditions from the three event conditions. We were more interested in examining the semantic influence of sound on visual event discrimination rather than the influence of differences in visual saliency of the three event videos. A one-way, repeated measures ANOVA with the aggregated data (Figure 2) revealed a significant main effect of audiovisual soundtrack condition [*F*(2,26) = 10.263, *p* = 0.001, η^2^ = 0.275]. Planned contrast tests revealed that the RTs were significantly faster when participants concurrently heard a semantically congruent soundtrack in comparison to hearing a semantically incongruent soundtrack [*F*(1,27) = 13.273, *p* = 0.001, η^2^ = 0.330] or no soundtrack [*F*(1,27) = 12.710, *p* = 0.001, η^2^ = 0.320]. There was no significant difference between the RTs to discriminate the visual events when participants concurrently heard a semantically incongruent soundtrack in comparison to when no soundtrack was heard [*F*(1,27) = 0.106, *p* = 0.747, η^2^ = 0.004].

The results of Experiment 1 indicate that congruent auditory semantic information affects the time for dynamic visual events to gain access to visual awareness, and thus, suggest that semantic congruency-based audiovisual multisensory integration occurs while visual information is suppressed from visual awareness. The present results are consistent with a previous study showing that congruent semantic information contained within auditory soundtracks can enhance the perceptual dominance of dichoptically viewed images during binocular rivalry (Chen et al., 2011). These results suggest that the longer predominance due to semantic congruency during binocular rivalry (Chen et al., 2011) can result from a shortened suppression period due to multisensory information processing. The modulatory influence of auditory semantic context on unconscious visual processing further supports that purely semantic-based multisensory integration can happen regularly in everyday life situations.

## Experiment 2

What are the mechanisms that caused the semantic-based congruency effect observed in Experiment 1? Semantic priming is a plausible mechanism that can explain the early breakup of suppression for the congruent audiovisual events. Semantic priming can be observed when an enhancement of accuracy or reaction time in response to a target stimulus is due to the presentation of a semantically associated priming stimulus that precedes the presentation of a target stimulus (Dehaene et al., 1998; Costello et al., 2009). A target word suppressed from visual awareness by CFS breaks up suppression and is perceived earlier when a semantically congruent prime word is viewed prior to presentation of a target word with CFS, compared to when the prime word and target words are semantically incongruent (Costello et al., 2009). These results indicate that semantic congruency enhances the strength of a target stimulus and consequently the target breaks up suppression sooner. Recent studies suggest that crossmodal semantic priming of congruent naturalistic sounds presented prior to visual stimulus presentation can enhance visual sensitivity (Chen and Spence, 2011) and result in shorter reaction times to identify natural objects (Schneider et al., 2008).

Close temporal proximity of multiple unimodal sensory components has been shown to be important for multisensory integration (Meredith et al., 1987; van Atteveldt et al., 2007). We hypothesized that by presenting soundtracks prior to the discrimination of silent event videos, the potential influence of crossmodal semantic priming on participants’ visual awareness of the events in Experiment 1 can be assessed while limiting the influence of concurrent multisensory integration. If the semantic congruency effect is abolished by the prior presentation of sound, this result indicates that the facilitatory effect of semantic congruency observed in Experiment 1 may be caused by a different mechanism than crossmodal semantic priming.

### Methods

Fifty-one undergraduate students participated in Experiment 2 that did not participate in Experiment 1. All apparatuses and stimuli were identical to those used in Experiment 1, except that the onset and offset of auditory soundtrack presentation immediately preceded the onset of dichoptic Mondrian and target video presentation. Auditory soundtrack presentation in Experiment 2 always lasted for 3 s to allow adequate time for semantic information to be accessed prior to performance of a 3AFC video discrimination task that was identical to that done by participants in Experiment 1. Following the initial soundtrack presentation, the event videos were always presented silently, so all discrimination trials of Experiment 2 resembled the silent audiovisual condition trials of Experiment 1.

### Results and Discussion

**FIGURE 3:**
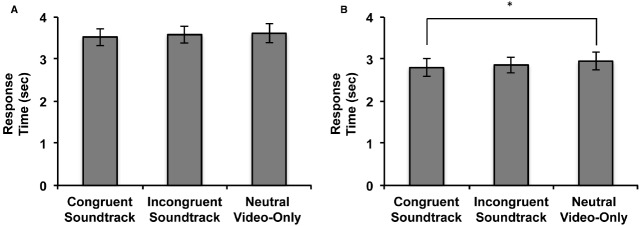
**Results of Experiment 2 and 3. (A)** RTs for the aggregated audiovisual soundtrack conditions in the 3AFC identification task when soundtracks were heard prior to silent event video viewing. **(B)** RTs for the aggregated audiovisual soundtrack conditions in the 3AFC identification task when soundtracks were heard during static image viewing (*p ≤0.05). Error bars represent ± 1 standard error.

The data screening procedure based on individuals’ error rate were identical to that used in Experiment 1. We excluded fourteen participants with greater than chance error rates leaving the data of 37 participants for analysis. A one-way repeated measures ANOVA conducted on the factor of audiovisual soundtrack condition, aggregated over three events, did not reveal a significant main effect on participants’ overall RTs to discriminate the visual events [*F*(2,35) = 1.319, *p* = 0.274, η^2^ = 0.035]. This result indicates that when a soundtrack is played prior to the visual event, auditory semantic congruency has no significant influence on interocular suppression durations. However, despite the lack of significant differences, the overall average RTs in Experiment 2 when comparing the congruent, incongruent, and the neutral video-only audiovisual soundtrack conditions does resemble the one observed in Experiment 1 (Figure 3A). This tendency indicates that crossmodal semantic priming may partially contribute to the audiovisual semantic congruency effect observed in Experiment 1, but the temporal concurrence of auditory and visual stimulus presentation may be the factor that determines whether the multi-sensory integration of semantic information can occur.

To further assess the possibility that the results of Experiment 1 can be explained by semantic priming, a mixed design ANOVA was conducted on the aggregate data from Experiment 1 and Experiment 2 with the audiovisual soundtrack condition as a within-subjects factor and the temporal relationship between auditory and visual presentation (concurrent audiovisual presentation for Experiment 1, and auditory prior to visual presentation for Experiment 2) as a between-subjects factor. The mixed design ANOVA revealed a significant interaction between the audiovisual soundtrack condition and the temporal relationship of audiovisual presentation [*F*(2,63) = 3.200, *p* = 0.044, η^2^ = 0.048]. This result further supports that crossmodal semantic priming cannot completely account for the facilitatory effect of audiovisual semantic congruency observed in Experiment 1.

## Experiment 3

It is possible that spatiotemporal crossmodal correspondences could have influenced the results observed in the congruent audiovisual soundtrack condition of Experiment 1. For example, there is a close temporal alignment of the finger movements of the guitar player seen in the guitar event video that occurred synchronously with the sounds of the guitar being played. As mentioned before, audiovisual temporal synchrony can shorten interocular suppression durations for dynamic talking faces (Alsius and Munhall, 2013; Plass et al., 2014). Thus, observers could have been influenced by temporal synchrony cues when discriminating the guitar video in the congruent audiovisual soundtrack condition instead of being influenced only by semantically congruent multisensory information.

Looming or receding auditory signals, which respectively refer to increases or decreases in sound intensity (Ghazanfar and Maier, 2009), could have corresponded with the movement of objects seen in the event videos and influenced the results observed in Experiment 1. Multisensory integration of auditory and visual stimuli can enhance behavioral performance in humans (Cappe et al., 2009). Looming and receding audiovisual correspondences could have been particularly relevant to the congruent audiovisual soundtrack conditions of the train and racecar events, because both events featured objects (an approaching train or circling racecars) that moved toward the perspective of the camera and then away in the case of the ending portion of the racecar event video. Additionally, a spatiotemporal correspondence related to the Doppler illusion may have influenced the results of Experiment 1. The Doppler illusion refers to an observer’s changing perception of pitch as a sound-emitting object in motion approaches and recedes relative to the location of an observer despite the unchanging frequency of the auditory signal emitted by a moving object (Neuhoff and McBeath, 1996). Specifically, the experience of the Doppler illusion includes a perceived gradual decrease in the pitch of the auditory signal emitted by a moving object as it approaches an observer followed by another quick decrease in perceived pitch as the moving object then passes the spatial location of the observer (Rosenblum et al., 1987). Thus, it is possible that audiovisual Doppler cues could have also served as a spatiotemporal audiovisual cue when discriminating the train and racecar event videos in Experiment 1.

To address the possibility that spatiotemporal crossmodal correspondences, rather than semantic congruency, may cause the facilitatory congruency effect observed in Experiment 1, we conducted an additional control experiment using static image event stimuli that eliminated the potential influence of residual spatiotemporal crossmodal correspondences on visual awareness. If participants discriminate static visual event images faster when hearing semantically congruent soundtracks in comparison to when hearing incongruent or no soundtracks, this would provide further support for the facilitatory effect of congruent audiovisual semantic information.

### Methods

Thirty-four undergraduate students who did not participate in Experiment 1 or 2 participated in Experiment 3. All apparatuses and stimuli were identical to those used in Experiment 1, except that the target stimuli used in Experiment 3 were static images that were selected from a single representative frame of each of the three target event videos used in Experiment 1 and 2.

### Results and Discussion

Identical data screening and aggregation procedures done prior to the analysis of data in Experiment 1 and 2 were done in Experiment 3. The data of seven subjects were excluded from analysis due to high error rates and the data from 27 participants were analyzed. A one-way repeated measures ANOVA conducted on the factor of audiovisual soundtrack condition revealed that there was a significant main effect on participants’ discrimination RTs [*F*(2,25) = 3.377, *p* = 0.042, η^2^ = 0.115]. Planned contrast tests between the soundtrack conditions revealed that RTs were significantly faster when participants heard soundtracks that were congruent with the suppressed event image viewed in comparison to when no sound was heard (Figure 3B) [*F*(1,26) = 6.500, *p* = 0.017, η^2^ = 0.200]. Unlike in Experiment 1, there was no significant difference between the reaction times when participants concurrently heard a semantically congruent soundtrack in comparison to when they heard incongruent soundtracks [*F*(1,26) = 1.091, *p* = 0.306, η^2^ = 0.040]. Consistent with Experiment 1, there was also no significant difference between participants’ RTs when they heard soundtracks that were incongruent in comparison to when nothing was heard [*F*(1,26) = 2.587 *p* = 0.120, η^2^ = 0.090].

The results of Experiment 3 further support that auditory semantic contexts can significantly influence the latency for suppressed static visual images to gain access to visual awareness. This result is consistent with Experiment 1, confirming that the beneficial effect of multisensory integration observed in Experiment 1 can be induced by a purely semantic congruency between auditory and visual stimuli. When considering the results of Experiments 1–3 together, our findings suggest that the multisensory integration of semantic information can occur even when static and dynamic visual events are suppressed from visual awareness, but temporal concurrence of auditory and visual stimulation is required for audiovisual semantic congruency effects to occur.

## General Discussion

In the current study, we demonstrated that semantically congruent auditory information accelerated the time for visually suppressed familiar and dynamic events to gain access to visual awareness, indicating enhanced visual processing due to semantic congruency. In a control experiment, no significant audiovisual semantic congruency effect was observed when the soundtracks were presented prior to the onset of visual event presentation, which indicates that crossmodal priming cannot completely explain the congruency effect. We also replicated the crossmodal semantic congruency effect with static images, in which any residual spatiotemporal correspondences between the auditory and visual stimuli were removed. These results suggest that crossmodal integration of congruent semantic information occurs even when visual stimuli are not consciously perceived.

### Unconscious Semantic Processing?

Unconscious processing of emotional information has been consistently supported by behavioral (Adams et al., 2010; Yang et al., 2010) and functional imaging (Morris et al., 1999; Pasley et al., 2004; Jiang and He, 2006) studies. However, results are mixed for other types of unconscious semantic processing, such as that involving the semantics of written words and category-specific object information. Some behavioral studies show that interocularly suppressed words cannot induce semantic priming effects (Blake, 1988; Cave et al., 1998) and that high-level object adaptation is abolished if visual stimuli are rendered invisible during binocular suppression (Moradi et al., 2005). These results indicate that high-level semantic processing does not occur when visual stimuli are suppressed from visual awareness. Supporting this notion, human brain imaging studies show that object representation is eliminated during binocular rivalry suppression in inferior temporal cortex (Tong et al., 1998; Pasley et al., 2004). A recent ERP study also reveals that the N400 component, an index of semantic information processing, is missing when participants are completely unaware of the meaning of dichoptically presented words (Kang et al., 2011).

There is, on the other hand, accumulating evidence supporting unconscious processing of semantic information. Chinese (Hebrew) words suppressed by CFS break up suppression faster than Hebrew (Chinese) words for Chinese (Hebrew) readers, indicating that the meaning of words are processed unconsciously and can influence access to visual awareness (Jiang et al., 2007). Priming of associated visual words can result in a faster breakup of suppression for visually presented words suppressed by CFS (Costello et al., 2009, but see also Lupyan and Ward, 2013). It is also shown that suppressed words can affect behavioral performance in a problem-solving task (Zabelina et al., 2013). Human brain imaging studies demonstrate that multi-voxel pattern analysis can extract category-specific object information even when objects are suppressed from visual awareness during CFS (rendering BOLD signals reduced close to baseline) in category-specific areas such as FFA and PPA (Sterzer et al., 2008) and other visual areas such as the lateral occipital area and the intra-parietal sulcus (Hesselmann and Malach, 2011). These results suggest that semantic information conveyed by visual objects can survive strong interocular suppression.

The current study demonstrates that interocularly suppressed dynamic events gain access to visual awareness faster when they are semantically congruent with sounds. Although indicating that audiovisual crossmodal integration occurs during visual suppression, our results do not necessarily indicate the unconscious processing of semantic information. The current study cannot determine whether crossmodal integration with invisible visual stimuli requires semantic processing of both auditory and visual information. It is possible that semantic processing of sound, which was clearly heard in the current study, may enhance visual processing of the suppressed event without unconscious visual semantic analysis. Further studies are required to clearly answer this question.

### Potential Mechanisms of the Crossmodal Semantic Congruency Effect

The semantic crossmodal congruency effect observed in the current study may not be caused by semantic priming as observed in an aforementioned study (Costello et al., 2009). Whereas semantic priming can occur when a prime precedes the presentation of target stimuli, we did not observe a significant semantic congruency effect when the sound was presented before the presentation of the target stimuli. This result indicates that multisensory integration based on semantic congruency may also require temporal proximity (Meredith et al., 1987; van Atteveldt et al., 2007). However, we observed a weak tendency toward a congruency effect, which suggests that semantic priming may be partially involved in the present crossmodal semantic congruency effect. Differences between the current study and previously mentioned studies (Costello et al., 2009; Lupyan and Ward, 2013) may explain why a congruency effect with temporal displacement was not presently observed. First, since our stimuli depicted dynamic events (e.g., an approaching train or circling racecars), concurrent presentation of information might be more important than previous studies using static images (written words or objects). However, we excluded this possible explanation with a control experiment using static images. Second, previous studies used lexical stimuli to induce a priming effect, but we used naturalistic non-speech sounds from the events. Word primes may activate greater amounts of information in a more extensive semantic network compared to the natural sounds of the individual events.

Hard-wired connections between primary sensory areas and multisensory areas, such as superior colliculus (SC) and posterior superior temporal sulcus (pSTS; Stein and Meredith, 1993; Stein, 1998; Noesselt et al., 2010) as well as between primary sensory cortices (Driver and Noesselt, 2008) have been suggested as underlying mechanisms for the beneficial effect of multisensory interaction based on spatial and temporal congruency. However, the neural mechanisms of purely semantic-based multisensory interaction are still not clear. A few recent brain-imaging studies suggest that inferior frontal cortex (IFC) and pSTS areas are activated differentially between semantically congruent and incongruent audiovisual stimuli (Belardinelli et al., 2004; Hein et al., 2007; Plank et al., 2012). However, enhancement and reduction in BOLD responses to semantically congruent vs. incongruent audiovisual stimuli vary depending on the brain areas, and the interpretations for the changes in these BOLD responses are still under debate. Although further studies are required to reveal underlying neural mechanisms for semantic-based multisensory integration, we speculate that multisensory cortical areas contribute to the semantic congruency effect observed in the current study.

## Conclusion

In the current study, we examined whether an audiovisual semantic congruency effect can occur even when visual stimuli are suppressed from visual awareness. In a series of experiments, we show that visual events suppressed by CFS gain preferential access to visual awareness only when semantically congruent sound is concurrently heard but not when the same sound is heard before visual event presentation. Our results suggest that first, semantic-based audiovisual integration can occur when visual stimuli are rendered invisible, and second, multisensory integration based on semantic congruency also requires temporal proximity.

### Conflict of Interest Statement

The authors declare that the research was conducted in the absence of any commercial or financial relationships that could be construed as a potential conflict of interest.
